# 完全胸腔镜下解剖性肺段切除术治疗早期肺癌的体会

**DOI:** 10.3779/j.issn.1009-3419.2018.02.04

**Published:** 2018-02-20

**Authors:** 少华 马, 天生 闫, 可毅 王, 京第 王, 金涛 宋, 通 王, 未 贺, 洁 白, 亮 金, 海龙 梁

**Affiliations:** 100191 北京, 北京大学第三医院胸外科 Department of Thoracic Surgery, Peking University Third Hospital, Beijing 100191, China

**Keywords:** 腹腔镜, 解剖性肺段切除术, 肺肿瘤, VATS, Segmentectomy, Lung neoplasms

## Abstract

**背景与目的:**

肺段切除较肺叶切除能够更多地保留健康肺组织，但其在早期肺癌根治性方面仍存在争议。本研究分析35例完全胸腔镜下解剖性肺段切除术临床病理资料并复习文献，探讨胸腔镜下肺段切除术在早期肺癌治疗中面临的问题。

**方法:**

回顾北京大学第三医院2013年5月-2017年7月单一手术组35例完全胸腔镜下肺段切除术患者的临床病理资料，观察术中及术后并发症等安全性指标及淋巴结清扫数目、转移情况，并将术后病理类型与术前影像类型比对分析。35例肺段切除术中男性11例，女性24例，平均年龄57.7岁。病灶位于右肺上叶者8例，右肺下叶者8例，左肺上叶者13例，左肺下叶者6例。计算机断层扫描（computed tomography, CT）影像学肿瘤最大径平均12.7 mm，肺门及纵隔淋巴结最大直径均小于10 mm，以磨玻璃成为主者23例，以实性成分为主者12例。

**结果:**

35例均顺利完成电视辅助胸腔镜手术（video-assisted thoracic surgery, VATS）解剖性肺段切除术，平均手术时间为153 min，出血量为51 mL。术后漏气10例，均未超过3天。健侧肺不张1例，乳糜胸1例。平均住院时间为6.1天。出院后30天内门诊复查未发生其他院外手术相关并发症。病理为转移瘤者2例，良性肺病8例，原发肺癌25例。25例原发肺癌中浸润性肺腺癌14例[CT以肺磨玻璃影（ground-glass opacity, GGO）为主者7例]，微小浸润腺癌4例（GGO为主者3例），原位腺癌6例（CT均为纯GGO），肺鳞癌1例（CT以实性成分为主）。25例肺癌平均切除淋巴结7.2枚，所有淋巴结无癌转移。

**结论:**

VATS解剖性肺段切除术技术上安全可靠，其在肺癌治疗中适应症需严格掌握，其优势仍需前瞻性随机对照实验来证实。

## Abstract (en)

**Background and objective:**

Segmentectomy can retains more healthy lung tissue than lobectomy, but it remains controversial in oncology for early stage lung cancer. The aim of this study is to discuss the problems of video-assisted thoracic surgery (VATS) segmentectomy in early stage lung cancer, by analyzing the clinical and pathological data of 35 cases and reviewing the literature.

**Methods:**

There were 35 patients who received segmentectomy by complete video-assisted thoracic surgery, from May 2013 to July 2017, in single operation group in the Third Hospital of Peking University. We analyzed the patient's clinical and pathological data, intraoperative and postoperative complications, lymph node number and metastasis its situation, and compared postoperative pathology and preoperative computed tomography (CT) imaging type. In 35 cases of segmentectomy, there were 11 males and 24 females, with an average age of 57.7 years old. The lesions located in the right upper lobe were 8 cases, in the right lower lobe were 8 cases, in the left upper lobe were 13 cases, in the left lower lobe were 6 cases. The mean maximum diameter of CT imaging was 12.7 mm, and the largest diameter of hilar and mediastinal lymph nodes was less than 10 mm. 23 of them were ground glass predominating and 12 were solid components predominating.

**Results:**

All 35 cases were successfully completed VATS anatomical segmentectomy. The average operation time was 153 minutes, the amount of bleeding was 51 mL. There were 10 cases of air leakage after operation, all of which were not more than 3 days. There was contralat-eral atelectasis in 1 case, chylothorax in 1 case. The average length of hospitalization was 6.1 days. There was no other complications outpatient related to surgery, in 30 days after discharge. The pathological changes were as follow, 2 cases of metastatic tumor, 8 cases of benign lung disease and 25 cases of primary lung cancer. In the 25 cases of primary lung cancer, there were 14 cases of invasive lung adenocarcinoma (7 cases were groundglassopacity (GGO) predominating in CT imaging), 4 cases of micro-invasive adenocarcinoma (3 cases were GGO predominating in CT imaging), 6 cases of adenocarcinoma in situ (all were pure GGO in CT imaging), 1 case of lung squamous cell carcinoma (mainly composed of solid in CT imaging). An average of 7.2 lymph nodes were removed in 25 cases of lung cancer, and all lymph nodes had no metastasis.

**Conclusion:**

VATS anatomical segmentectomy is technically safe and reliable, and the indications for lung cancer need to be strictly controlled. Its advantages still need to be confirmed by prospective randomized controlled trials.

近年来，肺癌的死亡率及发病率不断上升，高居恶性肿瘤之首，严重威胁人类的生命健康，外科手术仍然是肺癌有效的治疗手段。自1995年以来，肺叶切除术加纵隔淋巴结清扫一直被视为肺癌手术的金标准^[1]^。在心肺功能较差的患者或者T1期肺腺癌患者中，肺段切除术是否能够替代肺叶切除术而发挥其最大限度保留肺功能^[2]^的同时达到根治效果^[3]^这一两者兼顾的优势一直争议未休。随着胸腔镜（video-assisted thoracicsurgery, VATS）技术的发展成熟，VATS解剖性肺叶切除术已被推荐为肺癌的常规手术方式，但VATS解剖性肺段切除术并未被广泛普及。因此VATS解剖性肺段切除术在肺癌治疗中的技术安全性、肿瘤根治有效性及保留肺功能的优势等问题的多重验证仍需前瞻性多中心随机对照实验来证实^[4]^。本文对我院单一手术组35例VATS解剖性肺段切除术资料行回顾性分析体会，并对VATS肺段切除术治疗肺癌面临的问题做一文献复习。

## 1 资料与方法

### 1.1 一般资料

2013年5月-2017年7月北京大学第三医院胸外科单一手术组实施完全胸腔镜下肺段切除术35例。35例肺段切除术中男性11例，女性24例，平均年龄57.7岁。病灶位于右肺上叶者8例，右肺下叶者8例，左肺上叶者13例，左肺下叶者6例。CT影像学肿瘤最大径为5 mm-38 mm（平均12.7 mm），肺门及纵隔淋巴结最大直径均小于10 mm，以磨玻璃成为主者23例，以实性成分为主者12例。35例术前均无病理活检，除1例确诊为支气管扩张外，余34例均严格按照原发肺癌分期检查行肿瘤学评估。

### 1.2 手术方法

手术方式均为胸腔镜三孔法解剖性肺段切除术。上叶肺段切除取腋中线第3肋间20 mm操作孔1，腋前线第6/7肋间10 mm观察孔，腋后线第8/9肋间15 mm操作孔2；下叶肺段切除取腋中线第4肋间20 mm操作孔1，观察孔及操作孔2同上叶肺段切除。手术顺序基本为依次处理肺动脉→支气管→肺静脉→段间平面，亦有肺静脉→支气管/肺动脉→肺动脉/支气管→段间平面者，肺静脉及支气管均使用Endo-GIA处理，肺动脉采用Endo-GIA、Homo-lock或者丝线结扎处理。肺门淋巴结先行采样送冰冻病理，确认无癌转移后行肺段切除术。段间淋巴结以抓持法单独切除或连同预切除肺段en-block切除，叶间淋巴结、肺门淋巴结及纵隔淋巴结根据术中原发病灶冰冻病理结果行采样或清扫（原位腺癌及微小浸润腺癌者采样，浸润性腺癌者系统性清扫）。段间平面采用膨肺再萎陷法确定。术后按照VATS肺叶切除术常规管理。全组右肺上叶后段切除7例，右肺上叶尖段切除1例，右肺下叶背段切除6例，右肺下叶后外基地段切除1例，右肺下叶前基地段切除1例，左肺固有上叶切除6例，左肺上叶舌段切除7例，左肺下叶背段切除6例。

### 1.3 观察指标

术中观察手术时间、出血量、术中损伤等指标。术后观察持续咳血、漏气时间、切口感染、肺不张、支气管残端瘘等常见并发症。并记录淋巴结清扫数目、SICU住院日术后住院日、胸管拔除时间等指标。所有患者均在术后1个月内门诊复查，观察30天内是否发生院外并发症。

## 2 结果

35例均顺利完成手术，无中转肺叶切除及中转开胸者。平均手术时间为153 min（31 min-272 min），出血量为10 mL-200 mL，平均51 mL，无术中重要脏器及大血管损伤。术后无持续咳血发生；漏气10例，均未超过3天；无切口感染；1例发生健侧肺不张，予气管镜吸痰后肺复张；1例左肺固有上叶切除者术后乳糜胸，保守治疗21天出院；无支气管胸膜瘘发生。术后入住SICU者4例，胸管平均留置时间为4.9天（2天-21天），住院时间为3天-21天（平均6.1天）。出院后30天内门诊复查未发生其他院外手术相关并发症。术后病理为转移瘤者2例，良性肺病8例（支气管扩张1例，支气管腺瘤样增生1例，局灶性肺纤维化1例，慢性炎症3例，未分型者2例）；原发肺癌25例，其中浸润性肺腺癌14例[CT以磨玻璃影（ground-glass opacity, GGO）为主者7例]，微小浸润腺癌4例（CT以GGO为主者3例），原位腺癌6例（CT均为纯GGO），肺鳞癌1例（CT以实性成分为主）。25例原发肺癌中肿瘤最大径5 mm-25 mm（平均11.9 mm）；平均切除淋巴结7.2枚（4枚-20枚），所有淋巴结无癌转移，均为Ia期（表 1）。

**1:** 25例非小细胞肺癌病理与影像对照资料 Cilinical and pathological data of 25 cases non-small cell lung cancer

Pathology	Density（CT, GGO component）			Diameter（CT, mm）		
	GGO≥50%（*n*=16）	GGO＜50%（*n*=9）		≤10 mm（*n*=13）	＞10 mm且≤20 mm（*n*=12）	＞20 mm且≤30 mm（*n*=0）
AIS	6	0		4	2	0
MIA	3	1		3	1	0
ADC	7	7		6	8	0
SCC	0	1		0	1	0
AIS: Adenocarcinoma in situ; MIA: Minimally invasive adenocarcinoma; ADC: Invasive adenocarcinoma; SCC: Squamous cell carcinoma; GGO: Ground-glass opacity.						

## 3 讨论

### 3.1 解剖性肺段切除术治疗肺癌的适应症

解剖性肺段切除术早在1939年就被应用于肺外科^[5]^，其在最大限度切除病灶的同时，也最大限度保留了肺功能^[2]^，尤其适合于心肺功能较差、高龄的肺部良性疾病患者，即便是肺功能良好的良性肺病患者，其优势也显而易见。肺转移瘤深在肺实质内无法实施楔形切除时，肺段切除术也是最佳术式之一。但对于原发肺癌患者则面临是否能都达到肿瘤根治的诟病，一是系统性淋巴结清扫^[6]^，一是切缘距离^[1]^，两者均影响着肺癌的预后。

随着计算机断层扫描（computed tomography, CT）技术的广泛应用，肺部小结节尤其是非实性肺结节的检出率明显提高，意即早期肺癌检出率随之明显提高，与传统胸片比较，CT能够降低死亡风险近20%，归咎其因在于更多的早期肺癌获得了早期外科手术根治的机会^[7]^。而早期肺癌能够获得良好预后的原因又有赖于病理类型。国际肺癌学会（International Associate for the Study of Lung Cancer, IASLC）2011年将肺腺癌分为不典型腺瘤样增生、原位腺癌、微浸润腺癌和贴壁生长为主的肺浸润性腺癌，其中原位腺癌及微小浸润腺癌的5年生存率高达100%^[8]^。结合CT影像学特征，原位癌及微小浸润腺癌又以GGO为主要表现。本组25例肺癌中6例原位腺癌均为纯GGO，4例MIA中3例为纯GGO，但14例浸润性腺癌中纯GGO仅占50%（7例）。而这种以GGO为主要成分的肺癌肺门及纵隔淋巴结累及率在5%以下^[9]^，且淋巴结转移基本遵循肺内到肺外顺序转移的规律^[10]^，这是肺段切除术在早期肺癌得到根治的一个理论根据。25例原发肺癌术中均对肺门淋巴结先行采样冰冻活检，证实无转移后行肺段切除，术后清扫之淋巴结均转移，为Ia期。14例浸润性腺癌没有实施肺叶切除术的可能存在以下原因：肿物在脏层胸膜下但未累及脏层胸膜，可获得足够切缘；术中单肺通气氧和指数不佳；术中冰冻病理为贴壁样生长肺腺癌；术中肺段切除后冰冻病理未能确切汇报是否为浸润性腺癌。这是本组病例在选择肺段切除术指征上存在的不足，且已按照美国国立综合癌症网络（National Comprehensive Cancer Network, NCCN）指南规范了后续诊疗。

每个肺段均为指向肺门的椎体结构，对于外1/3肺外周带，直径≤2 cm时实施亚肺叶切除可获得足够的切缘。1997年，Kodama等^[11]^报道一项平均直径≤2.5 cm的T1N0非小细胞肺癌不同术式对照研究，肺段切除组5年生存率93%，和肺叶切除组相似，但局部复发率高于肺叶切除组（2.2% *vs* 1.3%）。2006年，日本一项T1N0早期肺癌不同术式的多中心非随机对照试验^[12]^显示，扩大肺段切除组与肺叶切除组在生存率方面无差异（89.6% *vs* 89.1%），局部复发率反而低于肺叶切除组（4.9% *vs* 6.9%）。由此可见，在获得足够切缘的前提下，肺段切除是一种可以替代肺叶切除治疗早期非小细胞肺癌的术式。但全球范围内的两项多中心随机对照实验（JCOG0802, CALGB140503）均尚未给出明确结论^[13, 14]^。

因此，虽然没有明确的结论，但目前依据临床实验结果，临床肺段切除术遵循以下指征：①心肺功能较差，第一秒用力呼气量（forced expiratory volume in first second，FEV_l_）占预计值百分比 < 50%，高龄（≥75岁），或伴有其他合并症而不能耐受肺叶切除者；发现不同肺叶内肿瘤需同期手术者；肺叶切除术后肿瘤复发者；②肿瘤位于肺外1/3，局限于单一肺段内，肿瘤最大径≤2 cm，胸部CT观察肿瘤倍增时间≥400 d；切缘距离 > 2 cm或切缘距离/肿瘤最大径比值> 1；③胸部CT磨玻璃样结节中GGO实性成分 > 50%；术中冰冻病理提示为不典型腺瘤样增生、肺原位腺癌、微小浸润性腺癌和贴壁生长为主的浸润性腺癌，切除肺段的边缘病理证实为阴性；胸部增强CT或^18^氟-脱氧葡萄糖（^18^F-FDG）正电子发射型计算机断层扫描显像（PET-CT）未提示纵隔和肺门淋巴结转移且术中肺门淋巴结采样冰冻病理为阴性^[15]^。

### 3.2 解剖性肺段切除对初学者面临的技术问题

实施亚肺叶切除时，尤其是楔形切除时，肺结节的定位尤为重要。各种定位方法层出不穷，优劣尚难比较。但预实施肺段切除者，术前根据影像学已完全锁定结节所在肺段位置，其术中定位结节的目的在于确定结节与预切除段段间平面的距离，目前多采用CT三维重建集合定位丝/化学胶/弹簧圈等方法。本组病例定位均是以CT三维成像法确定结节位置，术后大体标本切缘均≥2 cm。但由于属开展此项工作的早期，本组选择的均是优势肺段，其中固有上叶、舌段及下叶背段切除者共计32例，仅3例为非优势肺段，分别为右肺上叶尖段、右肺下叶后外基地段及前基地段。优势肺段解剖相对简单，脉管系统较早独立于主干，切除方法等同于肺叶切除，术中肺膨胀再萎陷时也较容易确定段间平面。对于非优势肺段，陈亮提出的“锥式肺段切除术”值得肯定及学习^[16]^。其技术涵盖了准确判断和处理靶段支气管和血管以及解剖性分离段间交界面，可以实现精准的肺段切除甚至亚段及联合亚段切除。该方法要求术者对肺内脉管系统解剖相当熟悉，沿着脉管鞘尽可能向远端分离而实现精确的肺段间解剖。另当肺膨胀萎陷法不能很好的确定段间平面时，吲哚箐绿荧光染色可谓一种较好的方法^[17]^。

### 3.3 不同于肺叶切除术的常见并发症

本组35例肺段切除术未发生重大并发症，仅1例术后发生乳糜胸且保守治疗后痊愈，术中出血量、术后住院日、拔管时间等指标基本等同于肺叶切除，初步结果显示VATS肺段切除术是安全一种安全可靠的术式。但肺段切除与肺叶切除比较来看，有其特殊性，也就导致其不同于肺叶切除术后的特殊常见并发症。由于解剖性肺段切除术没有天然的肺裂，意即预切除平面没有完整的脏层胸膜包裹，因此，在实施“锥式肺段切除术”时，沿着脉管鞘分离至肺内时就会造成肺组织的破损而致术后持续漏气。术中可以使用修补材料如奈维覆盖创面，并使用生物蛋白胶喷洒在创面，如发现支气管损伤，必须缝合修补。术后处理方法：少量漏气可延长胸管引流时间，在数日内自愈；也可胸腔内注射粘连剂，如高糖、凝血酶、红霉素等，行胸膜固定；长期大量的漏气，要注意有无支气管胸膜瘘的存在，处理同支气管胸膜瘘^[16]^。本组术后持续漏气10例，术中试水均为肺表面破损漏气，未予修补，术后3日内漏气均停止。因肺段切除范围较小，术后胸膜残腔很快被过度膨胀的余肺填充，加之纤维素渗出，破损的肺短时间内会封闭，因此建议对肺表面破损不做过多修补以避免余肺膨胀受限。

术后咳血是肺段切除的另一个常见并发症，多来自于切缘渗血，予止血治疗多可缓解。但需注意术后肺淤血的发生。本组小样本35例术后均未发生进行性加重的咳血症状，考虑仍与选择优势肺段切除有关，其手术难度低，并发症发生率也较低。肺动脉及支气管走行相伴，但肺静脉却与肺动脉类似“插指”关系，在开展VATS肺段切除工作初期的经验不足，或血管变异时辨认不清，误断保留肺段的静脉，或者段间平面辨认不清而损伤段间静脉是术后“肺静脉梗死”的重要原因。肺淤血发生后主要以咳粉红色泡沫痰或鲜血且持续加重为特点，随之一旦出现无法解释的低氧血症及感染症状，应高度怀疑肺静脉损伤，确认“肺静脉梗死”时应当机立断二次手术切除淤血之余肺^[18]^。

本研究仅报告了35例VATS解剖性肺段切除术的初步结果和体会，尚难得出经验和结论，但结合文献复习认为VATS肺段切除术在技术上安全可行，可实现最大限度切除病灶及最大限度保留健康肺组织的双重优势，但仍需等待前瞻性随机对照实验结果进一步确认其在早期非小细胞肺癌治疗中的价值和地位。
